# Global Access to Radiotherapy—Work in Progress

**DOI:** 10.1200/GO.20.00562

**Published:** 2021-01-25

**Authors:** Mary Gospodarowicz

**Affiliations:** ^1^ Princess Margaret Cancer Center, Radiation Oncology, Toronto, Ontario, Canada

Cancer is one of the most challenging health problems in the world today. The challenges are multifactorial. First, the burden of cancer is already enormous with close to 19 million new cases expected in 2020, projected 10 million deaths, and the 5-year prevalence of cancer exceeded 43 million people in 2018. These numbers are expected to increase to 24.5 million new cancer cases in 2040 and 16.4 million deaths globally. Most of the growth in cancer cases is expected in low- and middle-income countries (LMIC), which are currently ill prepared to meet this challenge. In addition, cancer is one of the most complex health challenges. A single term cancer refers to hundreds of distinct disease entities, affecting every part of human body, any age, sex, and people all over the world. Cancer uses most health services ranging from the simplest to the most sophisticated.

Cancer is also one of the best documented diseases. Population-based cancer registration started in the Europe and the United States 80-95 years ago. Huge improvements in cancer screening, early detection, and treatment have been made, and now, more than 50% of patients survive 10 years or more in the United Kingdom^1^. However, there are huge disparities in cancer outcomes in the world with much lower survival rates observed in LMIC.^2^

Much has been written about the lack of access to cancer care globally.^3^ Among one of the best documented gaps in care is the lack of access to radiotherapy. The Lancet Oncology Commission on Expanding Global Access to Radiotherapy as well as many other publications documented the size of the problem, calculated the cost of scaling-up the access, and modeled the potential for saving lives and contributing to the economic growth.^3^

Recent attention to the radiotherapy problem brought to light many efforts to help to remedy the situation. In the last few months, several papers addressed various initiatives that are desperately needed to be addressed to facilitate the progress.

Much has been written about the gaps in access in almost every part of the world. Cancer and radiotherapy have some advantages in the global health arena. First, the International Agency for Research on Cancer is a WHO research agency fully dedicated to cancer. Besides extensive research effort on cancer etiology and prevention, it also engages in epidemiology research and provides first-class data on cancer incidence, mortality, and prevalence all over the world. The International Atomic Energy Agency (IAEA), another United Nations agency, has radiotherapy as part of their human health portfolio. The IAEA maintains the Directory of Radiotherapy Centres database that registers all available radiotherapy equipment in the world.^4^ In addition, the IAEA provides extensive research, education, quality assurance, and advisory function for procurement of new equipment to LMIC.

However, despite all the resources and efforts, the progress in closing the gap in access to radiotherapy is extremely slow. In fact, if there is no major change in the current programs, the situation in some parts of the world, especially sub-Saharan Africa and parts of Asia, may even deteriorate with the growth in the cancer burden eclipsing the growth in resources available. The global cancer and radiotherapy community wants to help and is extremely frustrated by the lack of guidance and realistic opportunities to contribute their expertise and resources to change this sad reality.

There is continuing improvement in the gross national product of LMICs. With that being said, there will be investment in health care. What is needed is local leadership and expertise to leverage the developing economy and harness new resources for cancer control.

One of the avenues for realistic assistance is in education. Radiation oncology departments worldwide offered training opportunities to many LMIC trainees; however, training abroad is expensive and often leads to the ‘brain drain’ where LMIC specialists decide to remain in a high-income country and not returning to their community. Most professional societies offer education courses abroad but often the curriculum is driven by the high-income country experts and is not applicable to the LMIC situation.

There is an increasing awareness that to expand the access to radiotherapy, we must build local capacity. That means supporting education of radiation oncologists where they are, either through partnership or twinning arrangements or support through multi-institutional multidisciplinary tumor boards between organizations. Technology is available today to connect experts around the world and permit exchange of ideas, common problem-solving, and give free access to educational resources. Benjamin et al^5^ describe the frameworks for radiation oncology global health initiatives in US residency programs.^5^ It is clear that the tide of interest in global cancer is changing with a wave of interest on the part of the younger faculty and trainees.

A growing number of publications refer to the use of the information and communication technologies in education and in medical practice. Telemedicine has the potential to mitigate a number of barriers to enable more equitable access to education and health care such as time and distance barriers. In radiotherapy, where computerization and automation are pervasive, telemedicine has huge potential to overcome the barriers and facilitate a more rapid adoption of precision.

Hatcher et al's^6^ description of brachytherapy training of professionals in a number of LMIC published in this journal provides an excellent example of that can be done with minimal resources to scale up the capacity for modern radiotherapy delivery in LMIC.^6^ The authors provided online training to a group of physicists, radiation oncologists, and dosimetrists from cancer centers located in Egypt, Ghana, Iraq, Jordan, Nepal, Nigeria, Mozambique, and Zambia, showing positive educator and learner experience. There is a need for systematic application of online learning opportunities with evaluation of the short-term and long-term outcomes in terms of not only learning outcomes but also translation of this new capacity into improved access to state-of-the-art radiotherapy and improved outcomes in recipients’ environments.

What is of particular interest is engaging experts in LMIC in leading these efforts. In another paper, Mailhot et al^7^ describe an initiative to assist radiation oncologists in Latin America in gaining expertise in modern precision radiotherapy. Rather than imposing their own curriculum, they surveyed interested colleagues in Latin America regarding their needs, which enabled them to tailor the curriculum to their needs.^7^

There is a visible shift from north to south giving and paternalistic model of collaboration to the true demand-driven partnerships and focused assistance. There is also a need for capacity building in high-income countries to increase a cadre of professionals engaged in global health. Global health, and in particular global cancer, has been considered a hobby in many academic centers. Some well-meaning professionals engaged in not-for-profit nongovernmental organizations that provide assistance to LMIC, and others travelled to countries needing assistance offering their individual services. While such endeavors help individuals, they have no impact on building local capacity, influencing policy, and are not sustainable.

Fortunately, there is new interest in a more long-term career development in global health founded on appropriate training by engaging in systematic broad institutional or organizational initiatives that are built on partnership with local professionals and trust and focused on sustained engagement. What is needed is an academic career development pathway for young professionals backed up by faculty opportunities and resources.
